# Sub-conjunctival Operation for Cataract

**Published:** 1842-12

**Authors:** 


### SUB-CONJUNCTIVAL OPERATION FOR CATARACT.

The inordinate and ever-present desire for something new, is the curse and reproach of French surgery and medicine, more especially of the former. *La nouveaute d'une opinion, d'une decouverte,* is as irresistible in its attractions to the profession there, as a candle is to a moth. Read the following:

"M. Bernard, of Paris, has published the details of an operation for depression of a cataract, in which he had recourse to the subconjunctival proceeding. The patient, an aged female, was seated in a low chair, the upper eyelid raised by a strabismus levator, and the eye fixed by a hook passed into the sclerotica. The conjunctiva being then drawn upon by another hook, the needle was passed in until it reached the place of election in the sclerotica, when the conjunctival hook being withdrawn, the elasticity of the membrane prevented its interfering with the passage of the needle. The cataract was then depressed in the usual way, and the instrument withdrawn. On examining the eye afterwards, the wound in the conjunctiva could not be perceived until the patient was directed to look inwards, when a slight red point, twelve millimetres from the cornea, could be distinguished. The patient had neither pain nor other inconvenience after the operation, and recovered perfectly.—*Prov. Med. Jour.* from *Gaz. Medicale.*

"M. Bernard of Paris" would doubtless have been as profitably employed in whittling sticks with a penknife, Yankee fashion. And yet we should not be surprised, if, after having performed the above wonderful achievement, he exclaimed, in the fulness of his beatitude,

> Non omnis moriar: multaque pars mei

> Vitabit Libitinam. Usque ego posterâ

> Crescam laude recens,———

A subconjunctival method of depressing a cataract! Now, what end is proposed to be accomplished by it that is not attained by the ordinary method? Look, too, at the assemblage of instruments—a hook to hold the eyelids, a hook inserted into the sclerotic coat, a hook to catch up the mucous membrane! A man of genius unquestionably, this same "M. Bernard of Paris," if genius is evinced by the simplicity of means required to effect an end! truly, a most fingent man, if we may use a Carlyle-ism. Peradventure some would-be Roux, some embryo Lisfranc, some unfledged Velpeau, or some *veritable Bernard,* will ere long present us with a subcutaneous method of amputating a limb! It would be but a fitting cap to the climax of their absurdities. C.

